# The relationship between cognitive function and arterial partial pressure O_2_ in patients with COPD: A meta-analysis

**DOI:** 10.1097/MD.0000000000009599

**Published:** 2018-01-26

**Authors:** Xia-Hong Wen, Yan Li, Dong Han, Li Sun, Ping-Xiao Ren, Dan Ren

**Affiliations:** a The Second Department of Respiratory, Shaanxi Provincial People's Hospital; b Xi’an Medical University, Xi’an, Shaanxi, China.

**Keywords:** anoxia, chronic obstructive, cognition disorders, meta-analysis, pulmonary disease

## Abstract

**Background:**

The high incidence of cognition disorders in chronic obstructive pulmonary disease (COPD) patients represents a main focus in public health field recently. Thus, we tried to explore relationship between cognitive function and arterial partial pressure O_2_ (PaO_2_) in patients with COPD as assessed by Mini-mental State Examination (MMSE) and/or Montreal Cognitive Assessment (MoCA).

**Materials and methods:**

Medical and scientific literature databases, such as Web of Science, PubMed, Cochrane Library, China National Knowledge Infrastructure, and Wanfang Database, were searched independently by 2 reviewers until February 2016. Correlation coefficient (*r* or *r*_s_) values were obtained from each study, and 95% confidence intervals (CIs) were calculated using STATA12.0 software.

**Results:**

A total of 2049 studies were produced, and 9 of which were analyzed (714 participants) in the meta-analysis. The pooled *r* observed medium relationship for all selected studies (*r* = 0.405, 95% CI 0.31–0.55), and notable heterogeneity was also tested between studies (χ^2^ = 17.72, *P* = .023; I^2^ = 54.9%). After the sensitivity and subgroup analysis, the heterogeneity significantly decreased. Subgroup analysis showed that MMSE score was stronger correlation between PaO_2_ and cognitive function than MoCA score in the COPD patients. Begg test did not indicate potential risk of publication bias.

**Conclusions:**

There was a negative correlation between cognitive function and anoxia in patients with COPD, so it may be extremely essential to predict and improve the status of hypoxia in COPD patients.

## 1 Introduction

Chronic obstructive pulmonary disease (COPD) is now the preferred term for a condition that is characterized by persistent respiratory symptoms and irreversible, progressive airflow limitation. It is now well recognized that the pathophysiology of COPD is frequently associated with a wide range of comorbidities, which defined as COPD associated multiple systemic lesions.^[1,2]^ The damage of nervous system, mediated by hypoxia mainly due to the pulmonary disease or the comorbidities that adversely affect the brain (such as smoking), is one of the most important nonrespiratory manifestation.^[1,3,4]^ The impairment of cognition could aggravate mortality and disability in COPD patients.^[5,6]^ The prevalence of COPD associated cognitive impairment is accounted for a sizeable proportion of its comorbidities. A study acknowledges that there were 10.4% of COPD patients suffering from cognitive problem.^[5]^ Dodd et al^[7]^ have even claimed that only 3% of COPD patients possess perfectly cognitive capacity.

Cognitive dysfunction, defined as the decrease of cognitive ability weaker than expected for a certain age and educational level of the individual, includes mild cognitive impairment (MCI) and dementia. MCI is an intermediate stage between normal cognitive aging and dementia.^[8]^ But the mechanisms involved in COPD patients with cognitive impairment are not fully understood because of their complexity. Arterial partial pressure O_2_ (PaO_2_), by contrast, is cost most study efforts. Some studies have claimed that PaO_2_ is significantly correlated with cognition disorders in COPD patients.^[7,9]^ However, other studies hold that there is no correlation between cognitive function and PaO_2_ in COPD group.^[10,11]^

The primary goal of the meta-analysis was to explain the inconsistencies of all eligible studies and to explore possible relationship between cognitive impairment and PaO_2_ in patients with COPD as assessed by Mini-mental State Examination (MMSE) and/or Montreal Cognitive Assessment (MoCA).

## 2 Materials and methods

### 2.1 Literature-retrieval strategy

A comprehensive electronic data retrieval was performed in Web of Science, PubMed, Cochrane Library, China National Knowledge Infrastructure, and Wanfang Database until February 2016. COPD was searched in all databases using as syntax: “Pulmonary Disease, Chronic Obstructive” [Mesh] OR “Pulmonary Disease, Chronic Obstructive” [Title/Abstract] OR “Chronic Obstructive Pulmonary Disease” [Title/Abstract] OR “COPD” [Title/Abstract], and cognitive impairment were described as the following syntax: “Mild Cognitive Impairment” [Mesh] OR “Cognition Disorders” [Mesh] OR “Dementia” [Mesh] OR “Mild Cognitive Impairment” [Title/Abstract] OR “Cognition Disorders” [Title/Abstract] OR “Cognitive Impairment” [Title/Abstract] OR “Cognitive Deficit” [Title/Abstract] OR “Cognitive Defect” [Title/Abstract] OR “Cognitive Decline” [Title/Abstract] OR “Dementia” [Title/Abstract].

### 2.2 Inclusion and exclusion criteria

**Figure 1:**
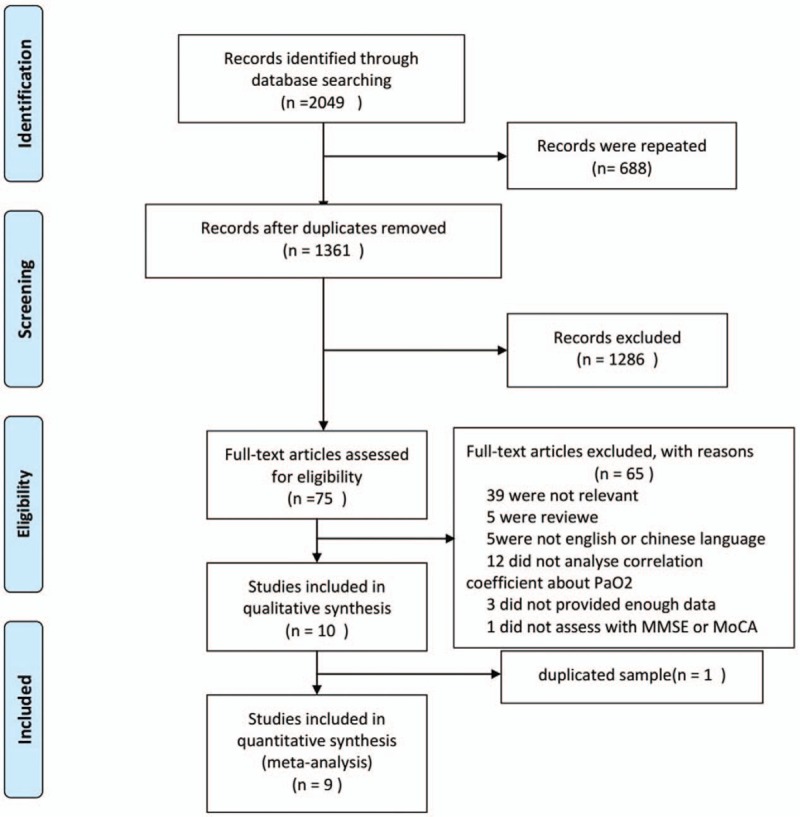
Flow diagram of studies selection process.

All the following criteria had to be met in the process. The inclusion criteria were as follows: The outcome was the association between PaO_2_ and cognition in patients with COPD; subjects were impaired cognition from COPD patients not with Alzheimer disease, vascular dementia, or other diseases that known to affect cognitive status, in order to ensure that cognition disorder was caused only by COPD; the effect indicator was the Pearson correlation coefficient, which also could be transformed from Spearman rho; the assessment tools for cognition were limited to MMSE and/or MoCA; and the report was only published in English or Chinese. The following studies were excluded: the repeated report and the most informative version report were introduced into our study, with regard to multiple reports including the same study population. The inclusion and exclusion processes were shown in Fig. 1.

### 2.3 Data selection and quality assessment

This study did not involve human or animal experiments, and thus ethical approval was not necessary. The data selection and quality assessment were operated by 2 independent authors (X-HW and YL) in a standardized manner. We would discuss with a 3rd reviewer (LS) to resolve any disagreements which were arose until it reached a consensus in the process. The statistics of studies from the above-mentioned databases on the links between cognition disorders and PaO_2_ in COPD patients were extracted using Endnote X7 software.

We appraise the quality of 9 cross-sectional studies with the Agency for Healthcare Research and Quality consisted of 11 rating items. Articles scoring criteria as follows: “4–7” regarding moderate quality, and it was low quality when <4 points, exceeding 7 points was considered as high quality.

### 2.4 Statistical synthesis and analysis

In this meta-analysis, we used STATA12.0 software (Stata Corporation, College Station, TX) to conduct the statistical analysis. There was an interchange between Pearson correlation coefficients and Fisher z-value with the following formula.^[12]^ Eventually, the summary *r* value was obtained with the summary Fisher z-value in the review. 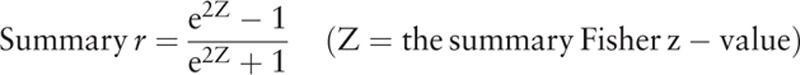


Correlation coefficient values were extracted from all computations, which were also transformed from Spearman rho.^[13]^ We regarded correlation effect sizes of 0.10, 0.30, and 0.50 as small, medium, and large correlation, respectively.^[14]^ Regarding the assessment of heterogeneity, the article was carried out using χ^2^-based Q testing and I^2^ statistics among studies, and statistical significance was set at *P* < .05.^[15]^ For significance of I^2^ values, we considered that the value of 25% to 50%, 50% to 75%, and 75% to 100% represented low, medium, and high heterogeneity, respectively.^[16]^ The random-effect model was adopted and subgroup analyses or meta-regression also was applied to analysis its source when heterogeneity existed obviously (I^2^ value > 50%). Otherwise, the fixed-effect model was used. Potential publication bias were checked by funnel plots^[12]^ and Begg test^[17]^ in this study, considered as statistically significant when *P* value < .05.

## 3 Results

### 3.1 Characteristics of included trials

A total of 2049 potential references were yielded with the original search of databases. After screening, 9 studies enrolling 714 participants met the inclusion criteria. The flow chart of selection of studies and reasons for exclusion were presented in Fig. 1.

As shown in Table 1, there was a detailed description among 9 trials, 7 of them used Pearson *r* values and 2 other papers utilized Spearman rho *r* values. The qualities of the included studies were all medium quality, also presented in Table 1.

**Table 1:**
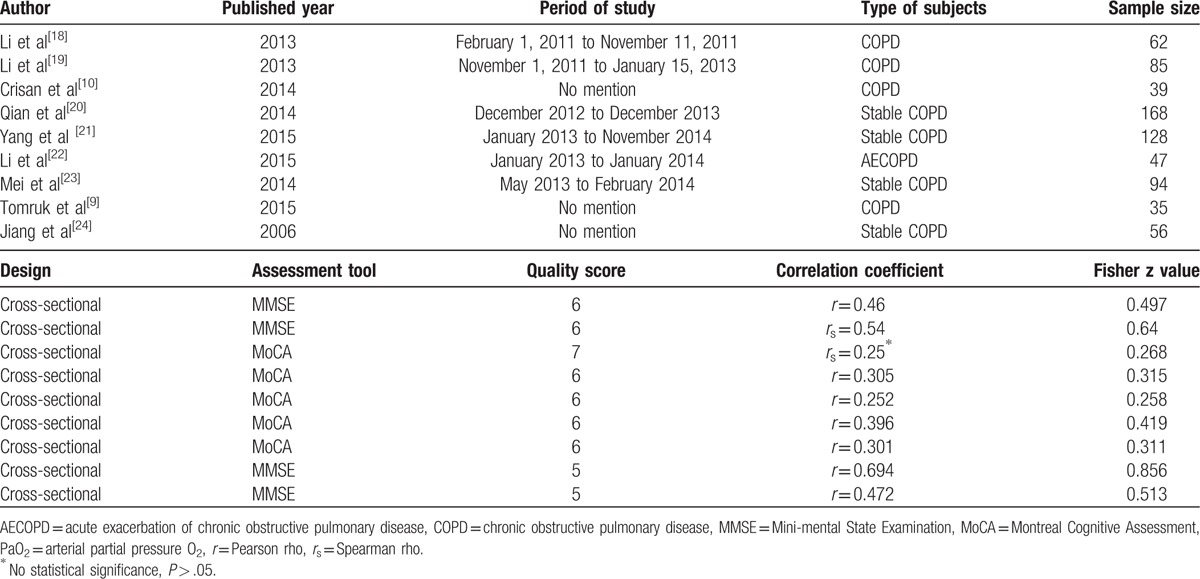
Baseline characteristics of trials included in meta-analysis.

### 3.2 The correlation between cognition and PaO_2_

**Figure 2:**
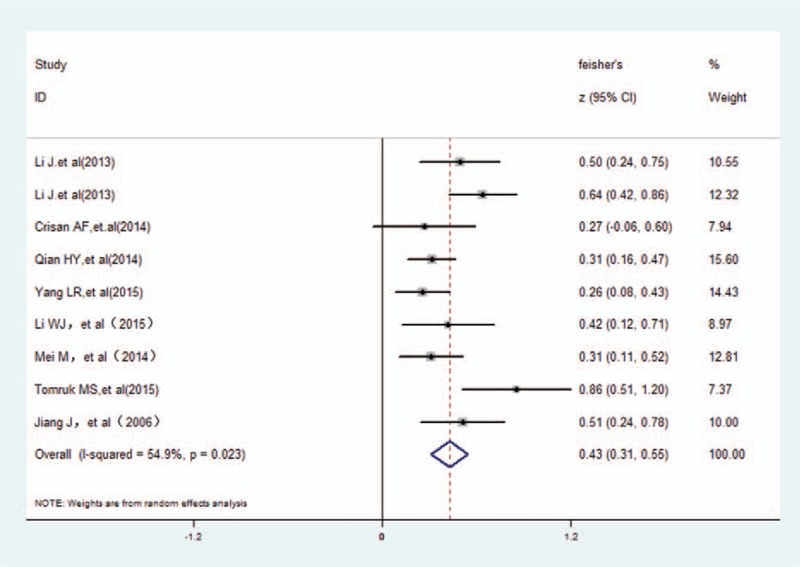
The association between cognition and PaO_2_. PaO_2_ = arterial partial pressure O_2_.

The study with COPD patients has claimed that PaO_2_ had a medium negative association with cognitive dysfunction (*r* = 0.405, 95% CI 0.31–0.55). However, the data indicated that there was high heterogeneity by random-effect model (χ^2^ = 17.72, *P* = .023; I^2^ = 54.9%) in Fig. 2.

### 3.3 Sensitivity analysis

Sensitivity analysis was conducted in comparison with significant heterogeneity across studies by deleting 1 single study each time from the pooled analysis. The result of analysis found that Li's^[19]^ and Tomruk's^[9]^ studies had a greater effect in the overall studies (χ^2^ = 12.46, *P* = .086, I^2^ = 43.8%; χ^2^ = 10.86, *P* = .145, I^2^ = 35.5%, respectively), indicating that they could affect the pooled Fisher z-value significantly.

### 3.4 Subgroup analysis

**Figure 3:**
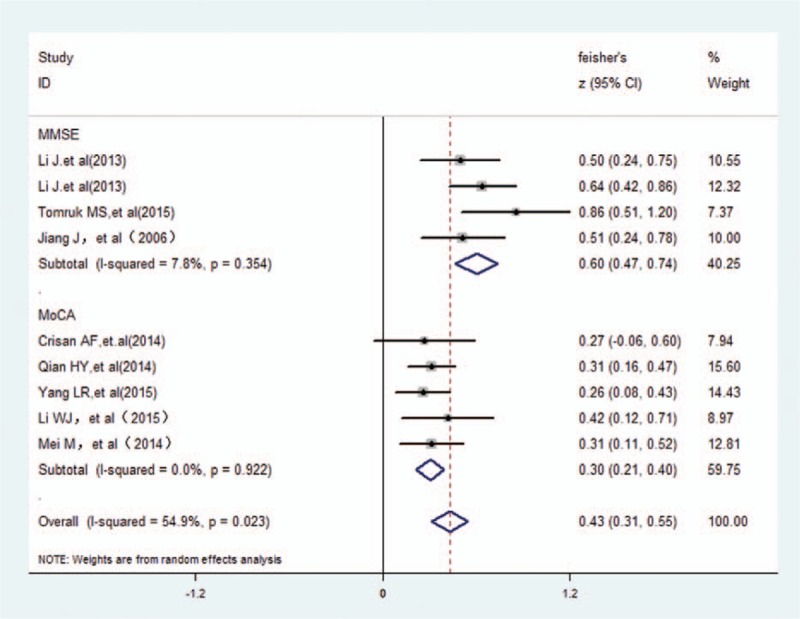
The association between cognition and PaO_2_ grouped by cognition assessment tool. PaO_2_ = arterial partial pressure O_2_.

**Figure 4:**
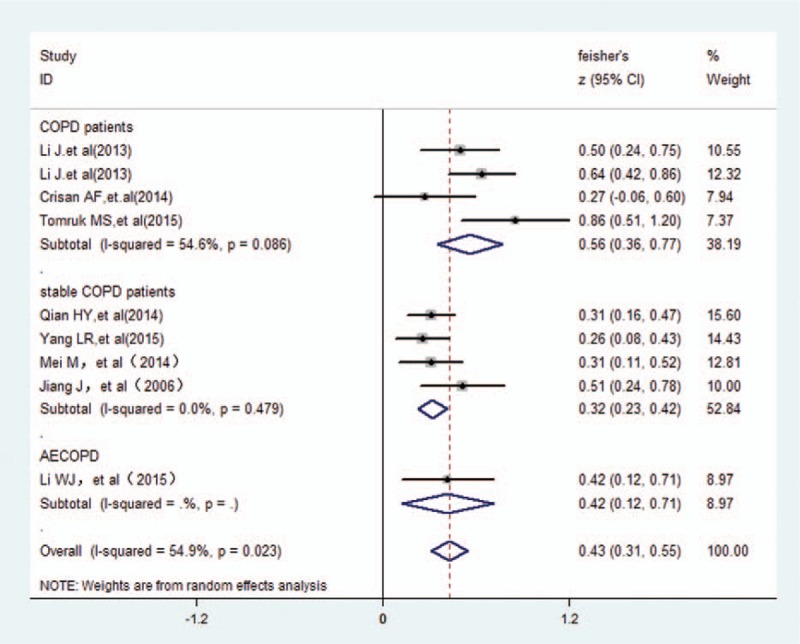
The association between cognition and PaO_2_ grouped by subject type. PaO_2_ = arterial partial pressure O_2_.

**Figure 5:**
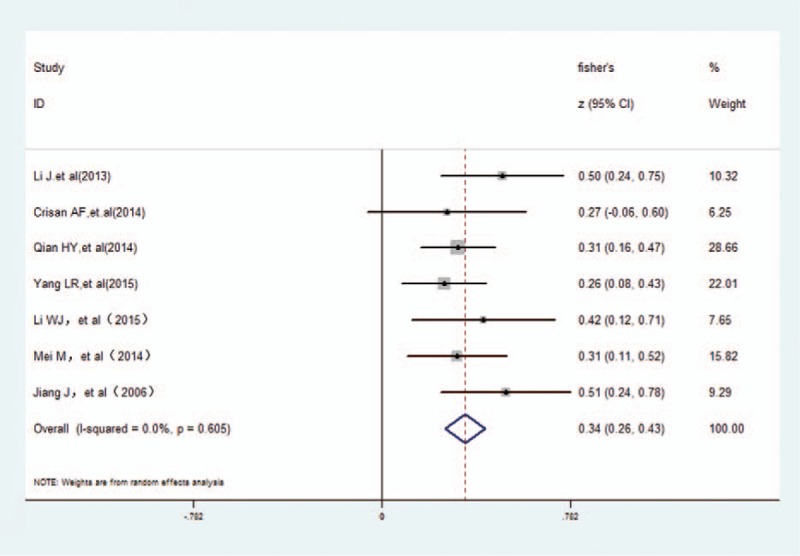
The association between cognition and PaO_2_ excluded Tomruk's and Li's studies. PaO_2_ = arterial partial pressure O_2_.

With regard to the links between cognition and PaO_2_ in COPD patients, we adopted subgroup analysis. According to the cognition assessment tool, there was 5 papers were assessed with an MoCA, revealing a small positive correlation (*r* = 0.291, 95% CI 0.21–0.40) and more closely when assessed by MMSE (*r* = 0.537, 95% CI 0.47–0.74) in Fig. 3. Regarding COPD states (stable disease and/or exacerbations), Fig. 4 indicated that there was a large correlation with COPD patients (*r* = 0.508, 95% CI 0.36–0.77), and medium for either groups of the stable COPD and acute exacerbation of COPD (*r* = 0.310, 95% CI 0.23–0.42; *r* = 0.397, 95% CI 0.12–0.71, respectively). After sensitivity analysis, there also was a medium positive correlation between cognitive function and PaO_2_ (*r* = 0.327, 95% CI: 0.26–0.43) when excluded 2 studies (Li's^[19]^ and Tomruk's^[9]^) in selected trials (Fig. 5).

### 3.5 Publication bias

**Figure 6:**
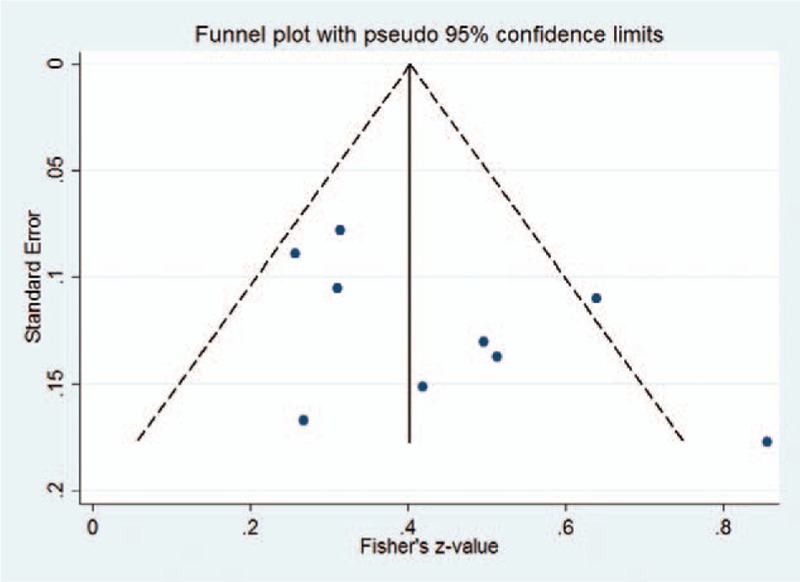
Funnel plot standard error by Fisher z-value.

As shown in Fig. 6, the funnel plot seemed to slightly asymmetrical based on visual inspection. The result of Begg test did not indicate potential risk of publication bias (z = 1.56, *P* = .118), which may result from the inclusion of the fewer relative studies.

## 4 Discussion

In the present meta-analysis, there are too few reviews to evaluate hypoxia COPD and associated cognition disorders in recent several years. It was evidence from our result that there was medium effect on cognitive decline in hypoxia COPD patients, which conformed with the results of other studies.^[10,18]^ Namely, the level of hypoxemia was positive correlated with the severity of cognitive impairment.

Until recently, intermittent and continuous hypoxia caused by COPD is recognized as a key mechanism that can adversely affect metabolism of neurotransmitters in the central nervous system.^[25,26]^ Among COPD patients especially in severe forms, the ischemia and subcortical atrophy are deteriorated mainly due to the decreased of cerebral blood perfusion. Eventually, cognition is impaired in COPD patients. Furthermore, Antonelli-Incalzi et al confirmed that the decline of drawing ability, one of the mainly detailed assessment regions, was highly beneficial to assessing the presence of cognitive impairment in hypoxemic COPD patients.^[5]^ The research found that low baseline oxygen saturation (<88%) was related to increased risk of cognitive impairment (OR 5.45; 95% CI 1.014–29.2; *P* = .048). Conversely, regular use of supplemental oxygen therapy decreased the risk for cognitive impairment (OR 0.14; 95% CI 0.07–0.27; *P* < .0001).^[25]^ Karamanli et al also thought the patients who got the long-term oxygen therapy might help a significant increase in cognitive status.^[27]^ To sum up, long-term oxygen therapy may be more beneficial in COPD patients.

However, a few studies found no statistically significant differences between hypoxemia and cognition disorder in COPD patients.^[10,11,28]^ Hung et al^[29]^ hold the patients with nonsevere COPD was not associated with worse cognition ability when compared with those without COPD, but severe COPD was associated with lower cognitive performance, which means that nonsevere COPD with or without hypoxemia could not arise directly the occurrence of cognitive impairment. And Liesker et al^[28]^ concluded that even nonhypoxemic patients with COPD showed significant impairments in cognitive performance.

The reason for the inconsistency might be poor understood about hypoxemia and associated cognitive performance. Based on prior research, it is still lacking a wide range of data with representation and universality from all over the word. Of course, studies differed in their composition of subjects should not be ignored, for example, assessment scale of cognitive capacity, demographic variables including age, education level, and the severity and staging of COPD and so on. Typically, the different scales with their characters were researched showing lots of controversies. Our meta-analysis revealed that MMSE score was more sensitive assessment scale than MoCA score (*r* = 0.537, 95% CI 0.47–0.74). However, another study implied that the MoCA score was superior to the MMSE score for the assessment of cognitive impairment.^[30]^ Dal Negro et al claimed cognition assessment, evaluated by 4 validated psychometric questionnaires including MMSE, the Clock Drawing test, Trail Making test A and B (TMT A and TMT B), would generate homologous but various correlation coefficients in patients with hypoxia COPD.^[11]^ And there were no statistically significant differences assessed by MMSE; on the contrary, the other 3 scales were more sensitive cognitive assessment tools with hypoxemic COPD patients.^[11]^

Besides, pulmonary function is indistinct for the link, some studies proved that cognitive impairment was related to the severity of COPD.^[30,31]^ However, other studies refuted that pulmonary function was not considered to be a reliable predictor for MCI in COPD populations.^[28,32]^ A study confirmed that the impairment of cognition was more severe in the late-stage COPD than mild-to-moderate disease, and there was irrelevant between cognition and the forced expiratory volume in 1 s (FEV_1_) in mild-to-moderate COPD patients, but both PaO_2_ and FEV_1_ could affect cognitive in severe patients.^[18,19]^ Furthermore, after the diagnosis of severe disease is established, higher partial carbon dioxide pressure, longer course, frequent exacerbation phenotype, or systemic inflammation, more number of exacerbation events in a year and so on will be taken in consideration to influence cognition.^[33]^ The evidence showed that patients with more frequent acute exacerbation events of COPD suffered worse cognitive function.^[34]^

This study has some limitations. First, data from the literature are still scarce, especially foreign research, and most studies are mainly clinical examinations with snail numbers of samples lacking of representation and universality about the link between hypoxemia and cognition in COPD group. Second, there is few data from the same study population utilize various questionnaires characterized by different sensitivities to measure their cognitive status, which could verify which one is best scale to assess the cognition. Third, publication bias might have existed even if it was not detected by Begg test, the reason is that our research with insufficient data only consisted of published studies, excluded unpublished studies.

## 5 Conclusion

In conclusion, the meta-analysis permitted that there was negative correlation between hypoxemia and cognitive function. Thus, it may be beneficial and imperative to predict and improve the status of hypoxia which could enhance the living quality of COPD patients.

## References

[1] Ozyemisci-Taskiran O Bozkurt SO Kokturk N Is there any association between cognitive status and functional capacity during exacerbation of chronic obstructive pulmonary disease? Chron Respir Dis 2015;12:247–55. 26071384 10.1177/1479972315589748

[2] de Oliveira JC de Carvalho Aguiar I de Oliveira Beloto AC Clinical significance in COPD patients followed in a real practice. Multidiscip Respir Med 2013;8:43. 23806051 10.1186/2049-6958-8-43 PMC3706245

[3] Greenlund KJ Liu Y Deokar AJ Association of chronic obstructive pulmonary disease with increased confusion or memory loss and functional limitations among adults in 21 states, 2011 behavioral risk factor surveillance system. Prev Chronic Dis 2016;13:E02. 26741996 10.5888/pcd13.150428 PMC4708003

[4] Dodd JW Lung disease as a determinant of cognitive decline and dementia. Alzheimers Res Ther 2015;7:32. 25798202 10.1186/s13195-015-0116-3 PMC4369069

[5] Antonelli-Incalzi R Corsonello A Pedone C Drawing impairment predicts mortality in severe COPD. Chest 2006;130:1687–94. 17166983 10.1378/chest.130.6.1687

[6] Eisner MD Iribarren C Blanc PD Development of disability in chronic obstructive pulmonary disease: beyond lung function. Thorax 2011;66:108–14. 21047868 10.1136/thx.2010.137661 PMC3111223

[7] Dodd JW Charlton RA van den Broek MD Cognitive dysfunction in patients hospitalized with acute exacerbation of COPD. Chest 2013;144:119–27. 23349026 10.1378/chest.12-2099

[8] China Prevention Expert Consensus Group of Cognitive Dysfunction. China expert consensus prevention of cognitive dysfunction. Chin J Geriatr 2006;25:485–7.

[9] Tomruk MS Ozalevli S Dizdar G Determination of the relationship between cognitive function and hand dexterity in patients with chronic obstructive pulmonary disease (COPD): a cross-sectional study. Physiother Theory Pract 2015;31:313–7. 25625565 10.3109/09593985.2015.1004768

[10] Crisan AF Oancea C Timar B Cognitive impairment in chronic obstructive pulmonary disease. PLoS ONE 2014;9:e102468. 25033379 10.1371/journal.pone.0102468 PMC4102489

[11] Dal Negro RW Bonadiman L Tognella S Extent and prevalence of cognitive dysfunction in chronic obstructive pulmonary disease, chronic non-obstructive bronchitis, and in asymptomatic smokers, compared to normal reference values. Int J Chron Obstruct Pulmon Dis 2014;9:675–83. 25061286 10.2147/COPD.S63485 PMC4085326

[12] Borenstein M Hedges LV Higgins JP Introduction to Meta-Analysis. New York, NY: John Wiley & Sons; 2011.

[13] Rupinski MT Dunlap WP Approximating Pearson product-moment correlations from Kendall's tau and Spearman's rho. Educ Psychol Meas 1996;56:419–29.

[14] Cohen J A power primer. Psychol Bull 1992;112:155–9. 19565683 10.1037//0033-2909.112.1.155

[15] Higgins JP Thompson SG Quantifying heterogeneity in a meta-analysis. Stat Med 2002;21:1539–58. 12111919 10.1002/sim.1186

[16] Higgins JP Thompson SG Deeks JJ Measuring inconsistency in meta-analyses. Bmj 2003;327:557–60. 12958120 10.1136/bmj.327.7414.557 PMC192859

[17] Begg CB Mazumdar M Operating characteristics of a rank correlation test for publication bias. Biometrics 1994;50:1088–101. 7786990

[18] Li J Huang Y Fei GH The evaluation of cognitive impairment and relevant factors in patients with chronic obstructive pulmonary disease. Respiration 2013;85:98–105. 23207572 10.1159/000342970

[19] Li J Fei GH The unique alterations of hippocampus and cognitive impairment in chronic obstructive pulmonary disease. Respir Res 2013;14:140. 24359080 10.1186/1465-9921-14-140 PMC3878035

[20] Qian H Lin H Li Y Assessment of cognition and associated factors in patients with stable chronic obstructive pulmonary disease. Zhonghua Jie He He Hu Xi Za Zhi 2014;37:769–73. 25537414

[21] Yang LR Li J Wei HM The relevant factors in patients with stable COPD. China J Modern Med 2015;25:75–9.

[22] Li WJ Wan YX Wang XP The occurrence and relevant factors in patients with acute exacerbation chronic obstructive pulmonary disease. Chongqing Med J 2015;44:2549–51.

[23] Mei M Luo H Wang TL Cognitive decline and relevant factors in patients with stable COPD. Med Information 2014;27:110–1.

[24] Jiang J Tian F Shao WW The evaluation of cognitive states in patients with stable chronic obstructive pulmonary disease. Chin J Prim Med Pharm 2006;13:2045–6.

[25] Thakur N Blanc PD Julian LJ COPD and cognitive impairment: the role of hypoxemia and oxygen therapy. Int J Chron Obstruct Pulmon Dis 2010;5:263–9. 20856825 10.2147/copd.s10684 PMC2939681

[26] Weuve J Glymour MM Hu H Forced expiratory volume in 1 second and cognitive aging in men. J Am Geriatr Soc 2011;59:1283–92. 21718272 10.1111/j.1532-5415.2011.03487.x PMC3758858

[27] Karamanli H Ilik F Kayhan F Assessment of cognitive impairment in long-term oxygen therapy-dependent COPD patients. Int J Chron Obstruct Pulmon Dis 2015;10:2087–94. 26491279 10.2147/COPD.S88326 PMC4598205

[28] Liesker JJW Postma DS Beukema RJ Cognitive performance in patients with COPD. Respir Med 2004;98:351–6. 15072176 10.1016/j.rmed.2003.11.004

[29] Hung WW Wisnivesky JP Siu AL Cognitive decline among patients with chronic obstructive pulmonary disease. Am J Respir Crit Care Med 2009;180:134–7. 19423714 10.1164/rccm.200902-0276OC

[30] Villeneuve S Pepin V Rahayel S Mild cognitive impairment in moderate to severe COPD: a preliminary study. Chest 2012;142:1516–23. 23364388 10.1378/chest.11-3035

[31] Schou L Ostergaard B Rasmussen LS Cognitive dysfunction in patients with chronic obstructive pulmonary disease—a systematic review. Respir Med 2012;106:1071–81. 22579108 10.1016/j.rmed.2012.03.013

[32] Dodd JW Getov SV Jones PW Cognitive function in COPD. Eur Respir J 2010;35:913–22. 20356988 10.1183/09031936.00125109

[33] Tulek B Atalay NB Yildirim G Cognitive function in chronic obstructive pulmonary disease: relationship to global initiative for chronic obstructive lung disease 2011 categories. Respirology 2014;19:873–80. 24935516 10.1111/resp.12333

[34] Liao WC Lin CL Chang SN The association between chronic obstructive pulmonary disease and dementia: a population-based retrospective cohort study. Eur J Neurol 2015;22:334–40. 25303726 10.1111/ene.12573

